# Afterlife matters

**DOI:** 10.1017/S1478951526101941

**Published:** 2026-02-25

**Authors:** Zhaohui Su

**Affiliations:** 1 School of Public Health, Southeast University, Nanjing, China; 2 Key Laboratory of Environmental Medicine Engineering, Ministry of Education, School of Public Health, Southeast University, Nanjing, China

## Afterlife matters

It costs 8 Chinese yuan,
Just over one US dollar,
The “Are You Dead Yet” app.
In a country where even AI tools
Opted for the free model,
Millions still paid to download it.

The app stands out in its simplicity —
Users tap its button each day,
To prove they’re alive, not dead;
And if no tap is made for two days straight,
An email is sent to the emergency contact.

Though the concept is hardly new —
Many apps offer similar things,
The existential angst it sees and soothes
Resonates nonetheless.
It makes us think, deeply and uncomfortably —
Who would be on our emergency list?
Who would handle our afterlife matters?
Who would cherish our belongings, so rich with meaning?
Who would tend to our decaying body with care and love?

It costs 8 Chinese yuan,
Just over one US dollar,
The “Are You Dead Yet” app.
Yet it costs so much more,
In time and reflection needed,
To learn who we can truly trust,
And what we really value,
In life and beyond.

## Data Availability

Data are available upon reasonable request.

